# Effectiveness and Safety of Four Aerobic Exercise Intensity Prescription Techniques in Rehabilitation Training for Patients with Coronary Heart Disease

**DOI:** 10.1155/2022/1647809

**Published:** 2022-07-15

**Authors:** Tao Chen, Huiying Zhu, Qingyuan Su

**Affiliations:** ^1^Department of Chronic Disease Management, The Second Affiliated Hospital of Guangzhou University of Chinese Medicine, Guangzhou 510120, Guangdong, China; ^2^Department of Cardiac Function, The Second Affiliated Hospital of Guangzhou University of Chinese Medicine, Guangzhou 510120, Guangdong, China

## Abstract

**Methods:**

A retrospective study was conducted on all patients with CHD who were admitted to CR and completed cardiopulmonary exercise tests (CPET) in Guangdong Hospital of traditional Chinese medicine. According to the risk stratification method of CHD, all participants were divided into three groups: low, moderate, and high risk. The training target heart rates (HRt) of each participant were calculated according to the formula of heart-rate-reserve (HRR), maximum-heart-rate (MHR), target-heart-rate (THR), and anaerobic threshold (AT) method provided in the guideline. Among them, the HRR method using the maximum-heart-rate obtained by the age formula was named “HRR method A,” and that using the actual measured peak heart rate was named “HRR method B.” For the three groups, the effectiveness and safety indexes at the target-heart-rate zone set by the different formulas above are counted and compared using CPET data.

**Results:**

A total of 324 patients were included in the analysis. There was no significant difference between the target-heart-rate set by the HRR method A and AT method among the three groups (*P* > 0.05). The mean value of HRt set by other methods was lower than the AT heart rate (*P* < 0.05). The HRt set by the THR method was close to the AT, while that set by the MHR method was the lowest. The frequency of patients whose HRt was set by the MHR method was lower than the AT one, which was the highest. None of the participants had serious adverse events. There were no risks of ECG abnormalities in the low- and moderate-risk groups. The HRR method A had the highest incidence of various risks of ECG abnormalities, while the MHR method had the lowest one, and the safety of the THR method is close to that of the AT method (*P* < 0.05).

**Conclusion:**

The heart rate calculated by HRR method A is more consistent with the actual AT. All four techniques are safe in low- and moderate-risk patients. In high-risk patients, using HRR method A has certain risks. It is recommended to use the MHR method for safety reasons, but its effectiveness is low. If considering both effectiveness and safety, the THR method can be conservatively selected at the beginning of the CR program.

## 1. Introduction

Coronary heart disease (CHD) is the single most common cause of death globally. The prevalence of coronary heart disease in China is estimated to be 11.0 million cases, and nearly 40% of all CVD-related deaths are due to CHD [1]. For CHD patients, cardiac rehabilitation (CR) is a comprehensive treatment process for which there is strong evidence for the benefits such as significantly reducing all-cause and cardiovascular mortality [2, 3]. The cornerstone of a CR program is exercise training [4, 5], as aerobic exercise is a core component of it. Prescribing a safe and effective aerobic exercise program in CR is critical to improving functional capacity [6].

Exercise intensity is a key indicator that determines the safety and effectiveness of aerobic exercise program [7]. For safety reasons, patients are usually recommended to exercise without excessive intensity to avoid cardiovascular events. However, if the intensity is not appropriate, the effectiveness would not reach the target of rehabilitation. The majority of guidelines on exercise training in CR recommend aerobic exercise prescription based on moderate intensity. Guidelines recommend that stratification of risk (low, moderate, or high) for exercise complications [8] should be carried out before training in CHD patients. For patients with different risk stratification, the monitoring recommendation level during exercise is different [9]. Individuals in moderate and high-risk groups are required to exercise with electrocardiography (ECG) monitoring, especially in the early CR exercise program. Patients in the low-risk group can exercise at home in the absence of monitoring. During exercise, ECG monitoring is not only for safety but also to ensure that the heart rate of patients can reach the target intensity preset by the exercise prescription.

However, recent data suggests that the distribution density of clinics with a CR unit in China is only 1.32 per 100 million population [10]. Most of these clinics are concentrated in the economically developed areas along the southeast coast. Even in these CR clinics, CHD patients rarely exercise with ECG monitoring.

Moreover, at the beginning of the CR program, leading guidelines recommend that the exercise prescriptions should be based on a graded exercise test (GXT) or cardiopulmonary exercise test (CPET) [11]. The exercise test provides accurate information such as anaerobic threshold (AT) that we use to build patient-specific exercise prescriptions. The oxygen uptake (VO2) at anaerobic threshold match 60% peak oxygen uptake (VO2peak). It is the best intensity to exercise at with an AT heart rate for CHD patients [12]. But recent evidence shows that only 30% of CR clinics perform these baseline exercise tests [13].

In the absence of a baseline exercise test, guidelines suggest some techniques for setting heart rate targets for exercise to match moderate intensity as feasible alternatives for exercise prescription. Commonly used indicators of intensity in these techniques are percentages of heart-rate-reserve (%HRR), percentages of max heart rate (%HRmax), and resting heart rate plus 20–30°bpm (RHR + 20–30) [14, 15]. In addition, Borg's rating of perceived exertion (RPE) 6–20 scale is suggested for use to set a target of 12–16 (moderate to hard) for subjective intensity monitoring of exercise. A target of 11–13 is usually suggested at the beginning seen as moderate intensity [16].

It is plausible that these techniques are safe alternatives, but there is a paucity of evidence. Clinicians often have concerns when using these methods to prescribe exercise, especially when the patient is likely to exercise without monitoring. It is important to compare the effectiveness and safety of these tests. Therefore, our aim was to determine if these techniques can effectively and safely inform exercise prescription for individuals with CHD. The first primary objective was to determine if the corresponding exercise intensities (expressed in heart rate) set by these methods match the anaerobic threshold (representing moderate intensity). The second primary objective was to determine the frequency of exercise-induced abnormalities (both ECG abnormalities and serious adverse events) at the intensities that were set by the above methods during aerobic exercise. The third objective was to determine if the effectiveness and safety differed between them.

## 2. Materials and Methods

This trial was a retrospective chart study and was approved by the Ethics Committee of Guangdong Provincial Hospital of Traditional Chinese Medicine (ethics number: ZE2022-090-01). The requirement for informed consent was waived because of the retrospective nature of this study.

### 2.1. Setting and Participants

Both the heart rate as a certain indicator of intensity and the exercise-induced ECG abnormalities require patients to exercise under ECG monitoring. But in reality, very few patients can meet the requirements. Therefore, as an alternative, we collected the data retrospectively from the results of CPET in CHD patients under different heart rates set by the intensity techniques (detailed below) to study.

#### 2.1.1. Case Inclusion Criteria

A. CHD is defined as any medical history (ECG is stable for more than 72 hours) of myocardial infarction (MI), previous revascularization procedure with percutaneous coronary intervention (PCI), coronary artery bypass graft (CABG), coronary angiography (CAG), or coronary computed tomographic angiography (CCTA) which shows that there was ≥1 coronary artery stenosis ≥50%, within 6 months [8].

B. post-CPET: individuals who had completed a cardiopulmonary exercise test at the Second affiliated hospital of Guangzhou University of Chinese medicine.

#### 2.1.2. Exclusion Criteria

Patients with missing CHD related medical records or CPET examination data were excluded.

According to the inclusion and exclusion criteria, a total of 324 patients who were inpatients or outpatients in this hospital between June 2018 and February 2022 were included in this retrospective study (*n* = 324).

### 2.2. Calculation of Heart Rate by Exercise Intensity Techniques

When building an aerobic exercise prescription in CR for CHD patients, it is ordinarily recommended to set it at a sustained moderate intensity by the guidelines. This intensity of control, in practice, is achieved by controlling the heart rate (HR) of patients during exercise. The HR corresponding to moderate intensity exercise is usually calculated at 60%–80% of HR reserve (HRR), or 50%–70% of VO2 reserve, or HR at the AT.

The guideline provides four techniques for the actual calculation of exercise prescription: the heart-rate-reserve (HRR) method, max-heart-rate (MHR) method, target-heart-rate (THR) method, and the anaerobic threshold (AT) method to set the target-heart-rate at moderate intensity [17]. The AT method needs to be measured by CPET. The detailed calculation methods of these techniques are as follows.

#### 2.2.1. HRR Method

Target heart rate = (HRmax—RHR) × exercise intensity% + RHR.

The intensity range is usually 60%–80%. According to the guidelines and expert consensus, 60% was used in this trial as the initial exercise intensity level. “RHR” means resting heart rate, and “HRmax” means maximum-heart-rate during exercise. There are two ways to set the HRmax. We use the formula (220—age) to calculate the first HRmax inferred from the patient's age. The second one is the peak heart rate actually measured in the exercise test (such as CPET). Therefore, we get two methods to set the target-heart-rate by the HRR method. We named the first method with calculated HRmax “HRR method A,” and the other one “HRR method B” for distinguishing.

#### 2.2.2. THR Method

Target heart rate = RHR + 20–30 bpm. We take the lower value 20 to calculate in this study as the initial exercise intensity level.

#### 2.2.3. MHR Method

Target heart rate = age inferred HRmax × exercise intensity = (220—age) × exercise intensity. The intensity range here is 50%–85%. According to the expert consensus [8], the initial exercise intensity level is set as the lower limit of the intensity value of the formula, and then the intensity is gradually increased as the physical fitness improves. Therefore, we take it as 50% in this trial.

#### 2.2.4. AT Method

Target heart rate = heart rate at the anaerobic threshold (HRAT).

In this study, four training heart rates were calculated using the above methods (HRR method A and B, THR method, and MHR method). Then, from the data of each patient's CPET, the indicators of safety and intensity for each patient under these four training HR were collected and compared with the HRAT.

### 2.3. Cardiopulmonary Exercise Test

CPET was performed on a cycle-ergometer (CS-200 Ergo-Spiro, SCHILLER, Baar, Switzerland) fitted with a facemask for all subjects. Testing was done with expired gas analysis under continuous ECG monitoring. In order to terminate the test after 10 minutes, the load incremental phase of the test protocol followed an increasing work rate of 15–30 W/min using a ramp-pattern.

Standard 12-lead ECGs were obtained after adequate skin preparation, at rest, each minute during exercise, and 5–6 minutes during the recovery phase. RHR was the heart rate at rest, and HRpeak was the heart rate at peak exercise. The heart rate at anaerobic threshold was termed HRAT. HRmax was the age-predicted maximum-heart-rate, estimated by 220-age. All the heart rate and ECG abnormalities during the test were recorded.

Oxygen uptake (VO2) and carbon dioxide output (VCO2) were measured breath by breath. Respiratory gas analysis will be performed using a metabolic cart (Cardiovit CS-200 Touch, SCHILLER, Baar, Switzerland). The highest value or the plateau of oxygen uptake was termed VO2peak. The value of oxygen uptake at an anaerobic threshold was termed VO2AT. These indicators were standardized by bodyweight (ml/kg/min). Metabolic equivalents (METs) were expressed each minute at rest, during exercise, and in the recovery phase.

Test termination criteria include symptoms (i.e., leg discomfort/fatigue, dyspnea, chest pain, or other), >2 mm of horizontal or downsloping ST segment depression, or a drop of systolic blood pressure >20 mmHg during progressive exercise, or sustained ventricular tachycardia (VT) and NSVT that interfered with hemodynamic stability.

### 2.4. Measures

The following measures were extracted from the patient health record and data from the results of CPET.

#### 2.4.1. Patient Profile

Participant characteristics were recorded, including age, gender, body mass index (BMI), history of MI/heart failure (HF), history of PCI/CABG, complete revascularization, medical history (hypertension, diabetes, dyslipidemia, carotid/cerebral arteriosclerosis, etc.), arrhythmia history such as complex ventricular arrhythmias (VA), history of sudden death, left ventricular ejection fraction (LVEF), functional reserve (i.e., maximum metabolic equivalents, max METs), and use of *β*-blocker medications.

#### 2.4.2. Risk Stratification for Exercise Complications

According to the risk stratification method for CHD patient exercise complications, all participants were divided into low-risk, moderate-risk, and high-risk groups [18] (see Table 1).

#### 2.4.3. Cardiopulmonary Exercise Test Results

The results of CPET reflect the patient's response to exercise. These indicators include RHR, HRAT, HRmax, HRpeak, %HRmax, HRR, VO2peak (and the percentage of the predicted value), peak Mets, VO2AT (and the percentage of the predicted value), exercise ECG results (i.e., positive, suspicious positive, negative, complex VA or other), exercise angina pectoris, and other symptoms. All these data were continuously monitored and qualitatively interpreted by an experienced cardiologist.

### 2.5. Comparison of Target-Heart-Rate

The intensity at AT is usually considered as a typical moderate exercise intensity and is recommended by the guidelines [16]. We calculated the target HR for training of each participant according to the above four calculation formulas (HRR method A and B, THR method, and MHR method) and then compared these HR with their own HRAT (less than, reach, or exceed) to evaluate whether exercise at these target HR can reach the AT intensity. For each method, we calculated the average target HR in the three risk groups, counted the frequency of patients who reached, less than, or exceeded the HRAT each group and compared the above indicators.

#### 2.5.1. Comparison of Safety Indicators

In this study, safety indicators include exercise-induced clinically relevant ECG abnormalities and serious adverse events. For each intensity technique, we counted the frequency of these two safety indicators in CPET data at each calculated target HR zone, including the total frequency in all participants as well as the frequency in the three risk groups. Then we compared the characteristics of the above safety indicators in different intensity calculation methods. These two safety indicators were defined as follows:

*(i) Exercise-Induced Clinically Relevant ECG Abnormalities*. Clinically relevant abnormalities were defined as exercise-induced changes on ECG during exercise at the target HR zone that would prohibit exercise beyond the intensity at which it occurred. It included exercise-induced horizontal or downsloping ST segment depression or elevation of ≥1 mm from baseline; complex VA (multiform ventricular premature beats of ≥3 in 10 beats); nonsustained ventricular tachycardia (VT); second- or third-degree atrioventricular block; ventricular fibrillation (VF)/VT; or bundle branch block. All ECGs in the CPET data were reviewed and interpreted by an experienced cardiologist. A second cardiologist reviewed all ECGs to verify the findings. Discrepancies in interpretation were resolved through discussion.

*(ii) Exercise-Induced Serious Adverse Events*. Serious adverse events are defined as events that lead to permanent or lasting change in function, disability, death, or potentially life-threatening events (e.g., MI, sustained VT, cardiac arrest, or a condition that requires cardiopulmonary resuscitation during exercise).

### 2.6. Data Analysis

All statistical analyses were performed with SPSS Statistics 23 (SPSS Inc., Chicago, IL, USA). Descriptive statistics were used to characterize the patients. Continuous variables were presented as means with standard deviation (SD). Categorical variables were expressed as counts (percentages). Frequency values were used to describe the prevalence of patients who exhibited exercise-induced ECG abnormalities or serious adverse events during exercise at each calculated target HR zone. Paired *t*-tests were used to compare the test results in each intensity calculation method with those of the AT method. Analysis of variance or chi-square tests were used to compare the participant characteristics and exercise test results, as appropriate, for those in the three risk groups. A *P* value of <0.05 was considered statistically significant for all analyses.

## 3. Results

### 3.1. Patient Profile

According to the above-mentioned risk stratification method of CHD, all 324 patients were divided into three groups. 232 cases matched any high-risk items and were classified into the high-risk group. 15 patients matched all low-risk items and were classified into the low-risk group. The remaining 77 cases were classified into the moderate-risk group.

In order to minimize the impact of sample size difference on the results, the measurement data between the three groups are analyzed by one-way ANOVA and the post hoc comparison (Scheffe test is used when the variance is homogeneous and Tamhane T2 test is used when the variance is uneven). The counting data among the groups are analyzed by the chi-square test.

The basic clinical data and medical history data of patients (*n* = 324) are shown in Tables 2 and 3. The numbers of patients with other diseases such as COPD, nephrosis, and atrial fibrillation are too small to be statistically significant and are not shown in table.

In a comparison among three groups, the mean age of patients in the high-risk group (64.07 ± 9.71 years) was higher than that in the moderate-risk group and low-risk group (*P* < 0.05).The sex ratio of the high-risk group was significantly different from that of the low-risk group and moderate-risk group (*P* < 0.05).

Other indicators with significant differences between groups are functional reserve, HF, incomplete revascularization, and complex VA (*P* < 0.05). The differences are due to the fact that these indicators are items for grouping themselves.

### 3.2. Outcome Indicators of CPET

The main outcome indicators of CPET are shown in Table 4.

13 participants failed to measure the anaerobic threshold in the CPET, which reflects the poor aerobic capacity of these 13 patients. Most of the other participants had normal VO2AT oxygen uptake (228, 70.4%). The measured mean HRAT, metabolic equivalent at (ATMETs), measured VO2AT, and the percentage of measured VO2AT to predicted value were all in line with the normal level of adults.

The indicators as VO2AT, ATMETs, and the percentage of VO2AT to the predicted value were the highest in the low-risk group and the lowest in the high-risk group (*P* < 0.05), while there was no difference among the three groups in the mean value of measured HRAT (*P* > 0.05).

It is suggested that although the HRAT level is similar, the VO2 and METS of patients in the high-risk group were significantly lower than those in the low-risk group and moderate-risk group.

At the peak exercise level, there were significant differences in VO2peak, peak METs, and the percentage of VO2peak in the predicted value among the three groups (*P* < 0.05). The average VO2peak in the low-risk group was 28.3 ± 2.6 ml/kg/min, reaching 94.3% of the predicted value, which was the level of normal adults. The VO2peak decreased in moderate- and high-risk group. The mean value of VO2peak in the high-risk group was lower than that in the moderate-risk group (*P* < 0.05). The HRpeak of the high-risk group was 121 ± 21.1 bpm, which was the lowest in three groups (*P* < 0.05).

There was a difference in the HRmax calculated according to age (*P* < 0.05). This result should be related to the significant difference in age among the groups. There was no significant difference in RHR among the three groups.

The significant differences between groups in other indicators like Complex VA, ECG positive reaction, and the number of patients with normal VO2peak are due to the fact that these indicators are items for grouping themselves.

### 3.3. Effectiveness Indicators

The comparison of effectiveness indicators is shown in Table 5.

A paired *t*-test was performed between every HR calculated by the other 4 methods and the measured HRAT. Except for HRR method A, there was a significant difference (*P* < 0.05). This shows that the HR calculated by the HRR method A is consistent with HRAT.

In comparison among the three groups, the HR calculated by the HRR method B and the MHR method were significantly different (*P* < 0.05). And post hoc comparison showed a difference between moderate- and high-risk group in the MHR method (*P* < 0.05). According to Spearman's rank correlation coefficient, there is a significant correlation between this difference and both age and gender (*P*=0.00).

The frequencies of the participants whose target HRs were calculated lower than the HRAT were more than 50% in all four methods. Among them, the number (307, 94.8%) in the MHR method is the highest, followed by 268 (82.7%) in the HRR method B, and 208 (64.2%) in the THR method. The frequency of this type of patients in the HRR method A was the least at 164 (50.6%). There was no significant difference among the groups (*P* > 0.05).

From the above results, it can be seen that the HRR method A is most consistent with the measured HRAT. The average HR of the other three methods is lower than the measured one at. The order of the average HR from high to low is the THR method, HRR method B, and MHR method. The target HR of the THR method is closer to the measured HRAT except for HRR method A.

### 3.4. Safety Indicators

All participants had no serious adverse events occur through the motion test terminal. The indicator of exercise-induced horizontal or downsloping ST segment depression or elevation of ≥1 mm from baseline actually refers to the ECG positive reaction of an exercise load test. We found that the risk relevant ECG abnormalities in this study were mainly ECG positive reactions and complex VA, including 28 (8.6%) positive reactions and 27 (8.3%) complex VA. In addition, 1 participant developed nonsustained ventricular tachycardia and 2 participants developed paroxysmal bundle branch block. All the above participants belong to the high-risk group, of which ECG positive reactions accounted for 12.1% and complex VA accounted for 11.6%. There were no risk-relevant ECG abnormalities that occured in the low-risk and moderate-risk groups.

For each participant with risk-relevant ECG abnormalities, we counted the frequency of these indicators in CPET data at each target HR zone calculated by the above intensity technique. The result is shown in Table 6.

The distribution of ECG abnormalities in the HR interval of the above four methods and the AT method is different (*P* < 0.05). The highest frequency of all kinds of risk of ECG abnormalities occurred in the HRR method A, followed by measured HRAT and THR methods, and the MHR method has the lowest risk of ECG abnormalities (*P* < 0.05). The safety of the THR method is close to that of the AT method. From the above results, we can see that the frequency of risk of ECG abnormalities is low overall, and the MHR method is safer than other techniques.

Most of the patients with complex VA during exercise had a history of hypertension, carotid atherosclerosis, and PCI. Most of them also had normal LVEF and anaerobic threshold and decreased functional reserve. One third of these patients had ventricular arrhythmia at rest (*P* < 0.05).

## 4. Discussion

The results of the first part of our study show that according to the CHD risk stratification method, most CHD patients will be classified into the moderate- and high-risk groups, and the average age and female composition ratio will increase in the high-risk group. The reason for this grouping result may only be that CHD patients with moderate- and high-risk account for a high proportion in our hospital. In the long term, it is necessary to further multicenter research and statistics on the composition ratio of each risk group in the actual clinical work.

The second part of statistical results on CPET showed that, although the VO2, METs, and the percentage of VO2 in the predicted value in the AT period of CHD patients decreased with the increase of risk grouping level (*P* < 0.05), the mean values of them were still at a normal level. This result shows that regardless of the risk group, AT level in most CHD patients is normal. The decrease of aerobic capacity in the high-risk group is mainly reflected in the peak period. HRAT of each group is at a similar level. This result proves that each risk groups can use the measured HRAT as the standard of HR for moderate intensity exercise.

The third part of the results is about the effectiveness. It is generally believed that exercise rehabilitation can achieve a better curative effect in the HR range consistent with HRAT, which represents the HR of moderate intensity exercise.

When comparing the effectiveness of the four methods, our results found that the HR calculated by the HRR method A had the highest coincidence with the measured HRAT (*P* < 0.05). The target HR calculated by other methods is significantly different from HRAT. The coincidence degree is THR method, HRR-method B, and MHR method from high to low. When calculated by the MHR method, more than 90% of CHD patients have a target HR lower than actual HRAT.

When comparing among risk groups, the difference in effectiveness results only appears in the HRR method B and the MHR method, and further pairwise comparison shows that there is only a difference in the MHR method between the moderate- and high-risk group. The formula of the MHR method takes age as a unique variable, so the difference is mainly due to the fact that the age of the high-risk group is larger than that of other groups. There is also a significant correlation between this difference and gender, which may be related to the change in gender composition ratio in the high-risk group. The above results show that in most cases, except for the MHR method, the effectiveness of these methods is less restricted by risk grouping.

Why is HRR method A calculated using age-inferred HRmax more consistent with HRAT than HRR method B using measured HRpeak? The reason may be that the HRAT is normal for most patients, but the measured HRpeak is reduced, as the results have shown. When the same HRR formula is used to calculate the target HR, if the measured HRpeak is used as a variable, the obtained target HR will be lower than the normal HRAT. The HRmax calculated by the age formula is within the range of the normal reference value. When the HRmax is used by the HRR method, the target HR is more consistent with the normal HRAT. This indicates that calculating the target HR using the HRR formula should be based on the expected normal HRmax.

The MHR method also uses the HRmax calculated by the formula. However, which is different from the HRR formula using two variables as HRmax and RHR, HRmax is the only variable in the formula of the MHR method. In fact, when actually measuring HRAT, the HR rise caused by exercise stimulation is also based on the RHR. Actual RHR is affected by different conditions, such as the use of *β*-blockers. When RHR is not taken into account, it will directly affect the consistency of calculated HR to HRAT. The formula of the HRR method includes the variable of actual RHR, so it can avoid this problem and be consistent with HRAT.

The fourth part of the results is about safety indicators. All participants had no serious adverse events, and the proportion of ECG abnormalities at various risks was less than 10%. There were even no ECG abnormalities in low- and moderate-risk patients. It shows that the overall safety of exercise in CHD patients is high.

Risk of ECG abnormalities only occurred in high-risk patients, of which ECG positive reactions accounted for 12.1% and complex VA accounted for 11.6%. We compare the target HR (calculated by the four methods) and HRAT with the HR range of the above risk of ECG abnormalities in CPET data. The results showed that even in high-risk patients, when exercising according to the target HR set by the above intensity techniques, the proportion of risk of ECG abnormalities was less than 8%.

And most of the abnormal ECG appeared within the HR range set by the HRR method A (e.g., the occurrence rate of complex VA was 7.8%), followed by the measured HRAT and THR method. The frequency of abnormal ECG in HRR method B and MHR method is awfully low. Therefore, from the perspective of safety, there is a higher risk of exercising with the HR set by the HRR method A. If you exercise with the HR calculated by the MHR method, the risk is the lowest.

In our study, compared with the four methods, HRR method A has the best effectiveness, but there are higher risks; the MHR method is the safest, but its effectiveness is low. Previous studies [11] suggested that in the absence of a CPET test baseline, the exercise intensity at the beginning of CR should be set to resting heart rate + 20–30 and the RPE target 11–14. Although it seems vague, it may provide a safe and effective starting point for most patients. The results of our study support this suggestion, and we believe that if we take into account both the effectiveness and safety, the compromise method may be the THR method (i.e., resting heart rate + 20–30).

We analyzed the condition characteristics of patients with risk of ECG abnormalities. Except that 1/3 of them had ventricular arrhythmia at rest (*P* < 0.05), no significant and characteristic indexes were found to indicate the occurrence of risk of ECG abnormalities. Therefore, when making exercise prescriptions, doctors still need to ponder the disease aspect.

Previous studies [19–21] have shown that the exercise risk of CHD patients is relatively low. Although exercise may trigger cardiovascular (CV) events, developing good exercise habits and CV health can significantly reduce this risk [22]. Previous studies [23] have reported that exercise-induced CV events usually occur in the early stages of participating in CR, which has no correlation with the amount of CR exercise, the level of professionals, and whether ECG monitoring during exercise [24]. The factors that increase the risk of CV events and death are poor compliance with exercise prescriptions [17].

Our data may support the following view: in the early stage of CR aerobic exercise for CHD, it is still necessary to divide patients into risk strata. In low-risk and moderate-risk patients, the four exercise intensity techniques recommended in the guidelines are usually safe. In the absence of a measured anaerobic threshold heart rate, the HRR method A is more effective. For patients with high-risk, if there is no condition to exercise under ECG monitoring, conservative exercise prescription (e.g., MHR or THR method) is recommended. To set the target-heart-rate, the MHR method is the safest, and the THR method is the most effective. Both methods need to be combined with the degree of perceived fatigue, signs or symptoms at the same time. We should emphasize that even though the target-heart-rate set by the MHR method is conservative, it does not require the patient to reach this intensity immediately. It should be done gradually based on the patient's actual physical fitness. Even so, for high-risk patients, when they have the condition to exercise under ECG monitoring, they still should be strongly recommended to ECG monitoring, which will help to minimize the risk of cardiac exercise rehabilitation.

The advantage of this retrospective study is that it records the heart rate changes of patients from rest, warm-up to peak intensity exercise through CPET, and can describe the exercise performance of patients with different intensity and heart rate level, so as to provide basis for the judgment of effectiveness and safety. However, it is worth noting that there may be differences in patients' heart rates and other reactions between the load-increasing exercise program commonly used in CPET and the continuous exercise with a specific load fixed in exercise rehabilitation training. It cannot be confirmed by the current data, and it still needs to be explained by prospective research.

In addition, there is no fixed training mode in the exercise program because many factors (including physical fitness, enthusiasm, and skeletal muscle constraints) will affect the progress speed of patients. The intensity shall be increased timely and moderately within the scope specified in the latest assessment based on the staff's observation and the patient's subjective response. It is equally important to consider psychoeducation for patients in CR to facilitate adherence to physical activity [25].

## 5. Conclusion

In conclusion, when patients with CHD participate in CR and exercise at the target-heart-rate formulated by the four exercise intensity techniques recommended in the guidelines, unconditionally measuring the anaerobic threshold heart rate, the heart rate calculated by HRR method A is more consistent with the actual AT. The proportion of risk of ECG abnormalities and serious adverse events is very low. All four techniques are safe in low- and moderate-risk patients. In high-risk patients, the use of HRR method A has certain risks. When it is impossible to exercise under ECG monitoring, it is recommended to use the MHR method to set the target-heart-rate. If both effectiveness and safety are considered, the THR method can be selected at the beginning of the CR program.

## Figures and Tables

**Table 1:** Risk stratification of cardiovascular events during exercise.

Item		Risk stratification		
		Low-risk	Moderate-risk	High-risk
Exercise test index	Angina	No	Maybe yes	Yes
	Asymptomatic, with myocardial ischemia and ECG changes	No	Maybe yes, ST segment down shift <2 mm	Yes, ST segment down shift ≥2 mm
	Other obvious discomfort symptoms (e.g., dyspnea, dizziness)	No	Maybe yes	Yes
	Complex ventricular arrhythmias	No	No	Yes
	Hemodynamic response	Normal	Normal	Abnormal
	Functional reserve	≥7METs	5.0–7.0 METs	≤5 METs

Nonexercise test index	LVEF	≥50%	40%–50%	<40%
	History of sudden death/sudden death	No	No	Yes
	Resting complex ventricular arrhythmias	No	No	Yes
	Complications of MI or revascularization	No	No	Yes
	Myocardial ischemia after MI or revascularization	No	No	Yes
	Congestive heart failure	No	No	Yes
	Clinical depression	No	No	Yes

All low-risk items match belong to low-risk groups; any high-risk item matches belong to high-risk group. ECG, electrocardiography. LVEF, left ventricular ejection fraction. MI, myocardial infarction.

**Table 2:** Basic clinical data of patients.

	All Patients (*n* = 324 )		Low-risk (*n* = 15)		Moderate-risk (*n* = 77)		High-risk (*n* = 232)		*P* ^#^	*P* ^a^	*P* ^b^	*P* ^c^
	No. of patients (%)	Mean (SD)	No. of patients (%)	Mean (SD)	No. of patients (%)	Mean (SD)	No. of patients (%)	Mean (SD)				
Age, y	324	62.8 (9.9)	15	57.3 (6.7)	77	60.0 (10.3)	232	64.1 (9.7)	0.00	0.63	0.04	0.01
Men	210 (64.8)		14 (93.3)		62 (80.5)		134 (57.8)		0.00	0.41	0.01	0.00
BMI,												
kg/m^2^	324	24.2 (3.4)	15	22.9 (2.0)	77	23.8 (2.6)	232	24.4 (3.6)	0.14	0.39	0.05	0.31
LVEF, %	324	65.9 (8.5)	15	66.9 (6.7)	77	65.9 (7.1)	232	65.8 (9.0)	0.89	0.91	0.89	1.00
Functional reserve, METs	324	5.1 (1.5)	15	8.1 (0.7)	77	6.1 (0.7)	232	4.6 (1.3)	0.00	0.00	0.00	0.00
*β*-blocker												
Medications	185 (57.1)		9 (60)		37 (48.1)		139 (59.9)		0.21	0.39	1.00	0.07

*P* ^#^, *P* value of comparison among three groups; *P*^a^, *P* value of comparison between low-risk and moderate-risk group; P^b^, *P* value of comparison between low-risk and high-risk group; *P*^c^, *P* value of comparison between moderate-risk and high-risk group. BMI, body mass index; LVEF, left ventricular ejection fraction.

**Table 3:** Medical history data of patients.

	All Patients (*n* = 324)	Low-risk (*n* = 15)	Moderate-risk (*n* = 77)	High-risk (*n* = 232)	*P* ^#^	*P* ^a^	*P* ^b^	*P* ^c^
	No. of patients (%)	No. of patients (%)	No. of patients (%)	No. of patients (%)				
Type of CHD								
MI	91 (28.1)	5 (33.3)	18 (23.4)	68 (29.3)	0.76	0.63	0.80	0.70
HF	26 (8.0)	0	0	26 (11.2)	0.00	—	0.38	0.00
post-PCI	213 (65.7)	12(80)	48 (62.3)	153 (65.9)	0.42	0.19	0.40	0.57
post-CABG	4 (1.2)	0	0	4 (1.7)	0.45	—	1.00	0.56
Incomplete revascularization	56 (17.3)	0	0	56 (24.1)	0.00	—	0.03	0.00
Complex VA	23 (7.1)	0	0	23 (9.9)	0.01	—	0.37	0.00
History of other diseases								
Hypertension	207 (63.9)	7 (46.7)	44 (57.1)	156 (67.2)	0.10	0.46	0.10	0.11
Diabetes	128 (39.5)	4 (26.7)	32 (41.6)	92 (39.7)	0.56	0.28	0.32	0.77
Dyslipidemia	107 (33)	3 (20)	25 (32.5)	79 (34.1)	0.53	0.51	0.40	0.80
Carotid atherosclerosis	153 (47.2)	6 (40)	40 (51.9)	107 (46.1)	0.57	0.40	0.65	0.38

*P* ^#^, *P* value of comparison among three groups; *P*^a^, *P* value of comparison between low-risk and moderate-risk group; *P*^b^, *P* value of comparison between low-risk and high-risk group; *P*^c^, *P* value of comparison between moderate-risk and high-risk group. CHD, coronary heart disease; MI, myocardial infarction; HF, heart failure; PCI, percutaneous coronary intervention; CABG, coronary artery bypass graft; VA, complex ventricular arrhythmia.

**Table 4:** The main outcome indicators of CPET.

	All Patients (*n* = 324)		Low-risk (*n* = 15)		Moderate-risk (*n* = 77)		High-risk (*n* = 232)		*P* ^#^	*P* ^a^	*P* ^b^	*P* ^c^
	No. of patients (%)	Mean (SD)	No. of patients (%)	Mean (SD)	No. of patients (%)	Mean (SD)	No. of patients (%)	Mean (SD)				
HRAT, bpm	311	98 (13.0)	15	103 (12.7)	77	99 (11.8)	219	97 (13.3)	0.19	0.59	0.28	0.58
ATMETs, METs	311	3.2 (0.7)	15	4.3 (0.7)	77	3.6 (0.6)	219	3.0 (0.7)	0.00	0.00	0.00	0.00
VO2AT, ml/kg/min	311	11.3 (2.5)	15	15.2 (2.3)	77	12.6 (2.0)	219	10.6 (2.3)	0.00	0.00	0.00	0.00
VO2AT/pred%, %	311	43.1 (13.6)	15	50.9 (13.3)	77	46.6 (10.2)	219	41.4 (14.3)	0.00	0.53	0.03	0.01
VO2peak, ml/kg/min	324	17.8 (5.3)	15	28.3 (2.6)	77	21.2 (2.8)	232	16.0 (4.7)	0.00	0.00	0.00	0.00
VO2peak/pred%, %	324	69.9 (17.4)	15	94.3 (18.8)	77	78.4 (13.3)	232	65.6 (16.2)	0.00	0.00	0.00	0.00
HRpeak, bpm	324	125 (20.9)	15	147 (14.8)	77	132 (15.9)	232	121 (21.1)	0.00	0.01	0.00	0.00
HRmax, bpm	324	157 (10.4)	15	163 (6.7)	77	160 (10.3)	232	156 (10.4)	0.00	0.65	0.06	0.02
RHR, bpm	324	73 (10.5)	15	71 (13.0)	77	73 (9.7)	232	73 (10.6)	0.62	0.59	0.28	0.58
Complex VA in test	27 (8.3)		0		0		27 (11.6)		0.00	-	0.38	0.00
ECG positive reaction	28 (8.6)		0		0		28 (12.1)		0.00	-	0.23	0.00
ECG suspicious positive reaction	42 (13.0)		0		13 (16.9)		29 (12.5)		0.19	0.12	0.23	0.33
With Normal VO2AT	228 (70.4)		12 (80)		59 (76.6)		157 (67.7)		0.23	1.00	0.48	0.14
With Normal VO2peak	69 (21.3)		12 (80)		26 (33.8)		31 (13.4)		0.00	0.00	0.00	0.00
With Roughly normal VO2peak	27 (8.3)		1 (6.7)		7 (9.1)		19 (8.2)		0.94	1.00	1.00	0.81

*P* ^#^, *P* value of comparison among three groups; *P*^a^, *P* value of comparison between low-risk and moderate-risk group; *P*^b^, *P* value of comparison between low-risk and high-risk group; *P*^c^, *P* value of comparison between moderate-risk and high-risk group. HRAT, heart rate at anaerobic threshold; ATMETS, metabolic equivalent at anaerobic threshold; VO2AT, oxygen uptake at anaerobic threshold; Pred, predicted value; VO2peak, oxygen uptake at peak; HR peak, heart rate at peak; HRmax, age-predicted maximum-heart-rate; RHR, resting heart rate; VA, ventricular arrhythmia; ECG, electrocardiography.

**Table 5:** The comparison of effectiveness indicators.

Intensity technique	All patients (*n* = 324)			Low-risk (*n* = 15)		Moderate-risk (*n* = 77)		High-risk (*n* = 232)		*P* ^#^	*P* ^a^	*P* ^b^	*P* ^c^
	No. of patients (%)	Mean (SD)	*P*,^*∗*^	No. of patients (%)	Mean (SD)	No. of patients (%)	Mean (SD)	No. of patients (%)	Mean (SD)				
Mean value of target-heart-rate set by exercise intensity techniques, (bpm)													
HRR-m A	324	98 (8.5)	0.45	15	99 (9.7)	77	99 (7.2)	232	98 (8.8)	0.77	1.00	0.96	0.79
HRR-m B	324	88 (11.0)	0.00	15	94 (10.5)	77	91 (9.5)	232	87 (11.3)	0.01	0.54	0.08	0.09
MHR-m	324	79 (5.2)	0.00	15	81 (3.4)	77	80 (5.1)	232	78 (5.2)	0.00	0.65	0.06	0.02
THR-m	324	93 (10.5)	0.00	15	91 (13.0)	77	93 (9.7)	232	93 (10.6)	0.62	0.90	0.73	0.81
AT-m	311	98 (13.0)		15	103 (12.7)	77	99 (11.8)	219	97 (13.3)	0.19	0.59	0.28	0.58

Frequencies of whose target HR lower than HRAT.													
HRR-m A	164 (50.6)			9 (60.0)		37 (48.1)		118 (50.9)		0.69	0.40	0.49	0.67
HRR-m B	268 (82.7)			11 (73.3)		66 (85.7)		191 (82.3)		0.49	0.42	0.60	0.49
MHR-m	307 (94.8)			14 (93.3)		72 (93.5)		221 (94.8)		0.81	1.00	1.00	0.76
THR-m	208 (64.2)			12 (80.0)		56 (72.7)		140 (60.3)		0.06	0.79	0.13	0.051

Frequencies of whose target HR higher than HRAT.													
HRR-m A	160 (49.4)			6 (40.0)		40 (51.9)		114 (49.1)		0.69	0.40	0.49	0.67
HRR-m B	53 (16.4)			4 (26.7)		11 (14.3)		38 (16.4)		0.50	0.42	0.50	0.66
MHR-m	16 (4.9)			1 (6.7)		5 (6.5)		10 (4.3)		0.71	1.00	0.51	0.64
THR-m	106 (32.7)			3 (20.0)		20 (26.0)		83 (35.8)		0.16	0.87	0.21	0.11

*P*,^*∗*^ , *P* value of comparison between each intensity technique and AT-m by paired *t*-test; *P*^#^, *P* value of comparison among three groups; *P*^a^, *P* value of comparison between low-risk and moderate-risk group; *P*^b^, *P* value of comparison between low-risk and high-risk group; *P*^c^, *P* value of comparison between moderate-risk and high-risk group. HRR-m A, heart-rate-reserve method A; HRR-m B, heart-rate-reserve method B; MHR-m, max-heart-rate method; THR-m: target-heart-rate method; AT-m: anaerobic threshold method.

**Table 6:** Comparison of safety indicators in intensity techniques.

	*a*. No. and Frequencies with ECG positive reaction			*b*. No. and Frequencies with Complex VA			*c*.	*f*.
	No. of patients	(%) of all high-risk patients (*n* = 232)	(%) in all ECG positive reaction patients	No. of patients	(%) of all high-risk patients (*n* = 232)	(%) in all Complex VA patients (*n* = 27)	No. of patients	No. of patients
HRR-m A	7	3.0	25	18	7.8	66.7	1	2
HRR-m B	1	0.4	3.6	9	3.9	33.3	0	1
MHR-m	0	0	0	5	2.2	18.5	1	1
THR-m	4	1.7	14.3	13	5.6	48.1	1	1
AT-m	2	0.9	7.1	14	6.0	51.9	1	2
P△		0.00	0.00		0.00	0.00		

*P* ^△^, *P* value of the consistency comparison among the five methods in the distribution of the safety indicators (using the nonparametric test of Related Samples—Cochran's Q test, ) in the high-risk group or all the people with this safety indicators. c, no. of patients with nonsustained ventricular tachycardia; f, no. of patients with bundle branch block; HRR-m A, heart-rate-reserve method A; HRR-m B, heart-rate-reserve method B; MHR-m, max-heart-rate method; THR-m, target-heart-rate method; AT-m, anaerobic threshold method. Among the above-mentioned patients with ECG abnormalities, most of them with positive reaction have a medical history of hypertension, normal LVEF, normal anaerobic threshold, decreased functional reserve, and most of them use *β*-blocker medications, but there was no significant difference compared with negative patients (*P* > 0.05).

## Data Availability

The data used to support the findings of this study are available from the corresponding author upon request.
